# Facilitating Memory for Novel Characters by Reducing Neural Repetition Suppression in the Left Fusiform Cortex

**DOI:** 10.1371/journal.pone.0013204

**Published:** 2010-10-06

**Authors:** Gui Xue, Leilei Mei, Chuansheng Chen, Zhong-Lin Lu, Russell A. Poldrack, Qi Dong

**Affiliations:** 1 State Key Laboratory of Cognitive Neuroscience and Learning, Beijing Normal University, Beijing, China; 2 Department of Psychology, University of Southern California, Los Angeles, California, United States of America; 3 Department of Psychology and Social Behavior, University of California Irvine, Irvine, California, United States of America; 4 Imaging Research Center and Departments of Psychology and Neurobiology, University of Texas, Austin, Texas, United States of America; National Institute of Mental Health, United States of America

## Abstract

**Background:**

The left midfusiform and adjacent regions have been implicated in processing and memorizing familiar words, yet its role in memorizing novel characters has not been well understood.

**Methodology/Principal Findings:**

Using functional MRI, the present study examined the hypothesis that the left midfusiform is also involved in memorizing novel characters and spaced learning could enhance the memory by enhancing the left midfusiform activity during learning. Nineteen native Chinese readers were scanned while memorizing the visual form of 120 Korean characters that were novel to the subjects. Each character was repeated four times during learning. Repetition suppression was manipulated by using two different repetition schedules: massed learning and spaced learning, pseudo-randomly mixed within the same scanning session. Under the massed learning condition, the four repetitions were consecutive (with a jittered inter-repetition interval to improve the design efficiency). Under the spaced learning condition, the four repetitions were interleaved with a minimal inter-repetition lag of 6 stimuli. Spaced learning significantly improved participants' performance during the recognition memory test administered one hour after the scan. Stronger left midfusiform and inferior temporal gyrus activities during learning (summed across four repetitions) were associated with better memory of the characters, based on both within- and cross-subjects analyses. Compared to massed learning, spaced learning significantly reduced neural repetition suppression and increased the overall activities in these regions, which were associated with better memory for novel characters.

**Conclusions/Significance:**

These results demonstrated a strong link between cortical activity in the left midfusiform and memory for novel characters, and thus challenge the visual word form area (VWFA) hypothesis. Our results also shed light on the neural mechanisms of the spacing effect in memorizing novel characters.

## Introduction

Mounting evidence from functional imaging, developmental, and lesion studies has emphasized the critical role of the left midfusiform cortex in fluent reading. Strong midfusiform activation has been observed during processing of words as compared to nonwords in both alphabetic and logographic writing systems [1], [2], [3]. The left midfusiform also becomes more involved in reading with increasing reading fluency [4], [5], [6] (also see [7] for a review). In contrast, dyslexic readers showed impaired functional activation in this region [8], [9], [10]. In addition, lesions that led to midfusiform cortex damage [11] or disconnection to the left midfusiform cortex [12], [13] resulted in impaired, letter-by-letter reading.

Although the specific role of the left midfusiform in fluent reading is still under debate [14], [15], [16], recent studies have implicated a particularly important role of the left midfusiform in processing and learning the visual form of new writing systems, especially visually complex logographic languages such as Chinese. Contrary to the hypothesis that the left midfusiform (y coordinate around −54) is specialized in the processing of familiar words (e.g., [14]), strong midfusiform activation was observed when native Chinese and English speakers processed novel scripts, such as Korean characters or Tibetan letters [14], [15], [16], [17], [18], [19], or when Italian subjects processed novel Greek words [20], or when non-Chinese speaking American subjects processed Chinese characters [21], [22]. When stronger responses to familiar words than to foreign writing were found, they were located in a more anterior region (y coordinate around −40) of the fusiform [18], [21], [22]. It has been shown that visual word training led to increased proficiency in identifying novel visual word forms [16], [18], [23], accompanied by decreased neural activation in the left midfusiform cortex [16], [18]. More importantly, it has been shown that the leftward lateralization of midfusiform activation during initial learning strongly predicted the outcome and long-term (six-month) retention of a two-week training regimen [24], [25], [26]. These results suggest that the left midfusiform plays an important role in learning new scripts.

To further elucidate the functional role of the left midfusiform, the present study tested three hypotheses regarding the association between midfusiform activation and processing and memorization of novel characters. First, we examined whether repeated presentations of novel characters were associated with reduced or increased neural activity in the left midfusiform cortex by monitoring brain activities with functional MRI. Existing studies have yielded mixed results. On the one hand, there is evidence that repeated presentations of novel objects lead to increased neural activation [27], which is consistent with the visual expertise hypothesis [28]. On the other hand, other studies have found that short-term repetitions lead to deceased neural activation, for both novel scripts [18] and faces [29]. Based on the latter results, we hypothesized that repeated exposure to novel characters would result in decreased activation in the left midfusiform, among other regions.

Second, we investigated whether activation of the left midfusiform during learning was also associated with long-term memory for novel characters, both within subjects (using a subsequent memory design) [30], [31] and across subjects (based on correlation analysis). Although many studies have revealed strong midfusiform activation when a novel script is being processed, the functional significance of the activity is not clear. One way to shed light on this issue is to investigate the connection between midfusiform activation and learning outcomes [24], [25], [26]. Whereas previous studies have found that midfusiform activation could predict subsequent episodic memory of familiar words [31], [32], [33], it is unclear whether such correlations would also be observed for novel characters. Based on results from previous subsequent memory studies on one-shot learning [30], [31], [32], we hypothesized that stronger midfusiform activation during learning (across repetitions) would be associated with better long-term memory both within and across individuals.

The third question we addressed in the present study was whether we could improve the memory for novel characters by using manipulations that would increase the midfusiform's activity during learning. The answer to this question would provide stronger evidence regarding the functional role of the midfusiform in processing and memorizing novel characters. The manipulation we used was the spaced learning paradigm. Behavioral studies have shown that better memory can be achieved by increasing the lag between repetitions (i.e., the spacing effect) and/or by changing the font of nonwords across repetitions [34], [35], [36]. One explanation of the spacing effect is the deficient processing hypothesis, which suggests that massed learning would reduce the processing level of the second and subsequent presentations of an item [37]. Several mechanisms could contribute to deficient processing, such as decreased voluntary attention [38], reduced voluntary rehearsal [39], and short-term perceptual priming [40]. Among them, short-term perceptual priming has been particularly proposed to account for the spacing effect in cued-memory tasks for unfamiliar stimuli. It is believed that stronger perceptual priming during massed presentation would lead to reduced perceptual processing of an item after the initial presentation, and hence worse performance in the cued-recognition test that relies on the retrieval of the structural-perceptual information of the item.

Although no neuroimaging study has examined the short-term perceptual priming hypothesis underlying the spacing effect in memorizing novel characters, several neuroimaging studies using familiar words as learning material have found that increasing the repetition lag can decrease neural repetition suppression [41], [42], [43], and enhance subsequent memory [33], [44]. However, the exact locus of the spacing effect varies across studies, probably due to the use of different study materials and encoding tasks. For example, in a recent fMRI study using the paired-associates task, Callan and Schweighofer [44] found that spaced learning significantly improved performance in a cued-recall task, which was accompanied by increased activation in the left frontal operculum (a region implicated in verbal rehearsal). Similarly, Wagner et al [33] found that spaced presentation of words was associated with stronger activation in the left inferior frontal gyrus (IFG) in a semantic judgment task and also with better recognition memory of the words. In a recent fMRI study [29], we used novel faces as learning material and found that, compared to massed learning, spaced learning significantly reduced repetition suppression in the bilateral fusiform cortex and enhanced participants' memory for novel faces. Nevertheless, a recent behavioral study failed to reveal any strong correlation between repetition priming and subsequent memory, either within or across subjects [45]. Still, another study found that stronger repetition suppression was associated with better recognition memory [46]. To address this discrepancy, we have proposed that it is important to control factors that could affect the amplitude of repetition priming, such as the variance in stimuli [29]. The use of novel characters with which subjects have no prior experience would help to reduce the variance. Considering the important role of the left midfusiform in processing novel scripts, we hypothesized that, compared to massed presentation, spaced presentation of novel characters would reduce repetition suppression in the left midfusiform cortex and improve subsequent memory.

## Methods

### Participants

Twenty subjects (9 males, mean age = 23.16±3.10 years, ranging from 19 to 30 years) participated in this study. All subjects had normal or corrected-to-normal vision and were strongly right-handed as judged by Snyder and Harris's handedness inventory [47]. None of them had a previous history of neurological or psychiatric diseases. None of them knew any Korean. Data from one subject were discarded due to a minor stomachache during the scan. Informed written consent was obtained from the subjects before the experiment. This study was approved by the Institutional Review Board of the State Key Laboratory of Cognitive Neuroscience and Learning at Beijing Normal University.

### Materials

**Figure 1:**
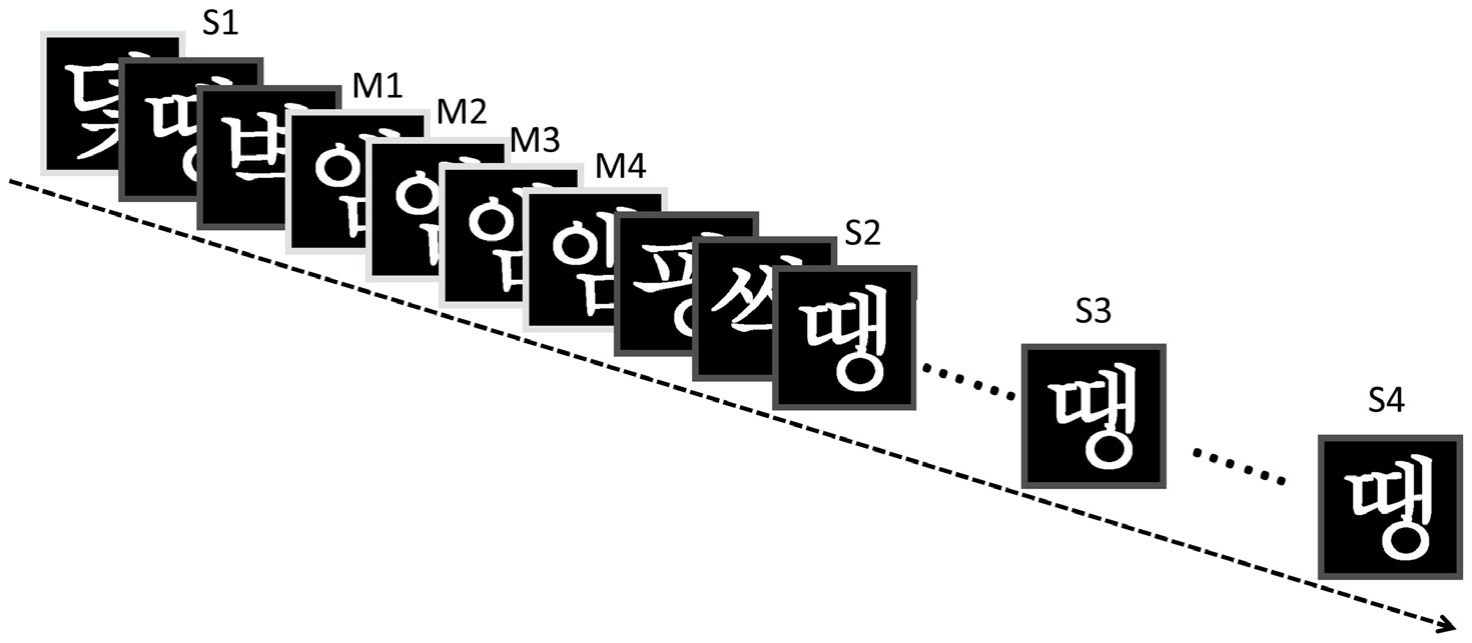
Experimental design. Each novel Korean character was repeated four times, consecutively for massed learning and in an interleaved manner (with an inter-repetition interval ranging from 6 to 20 trials) for spaced learning. Each stimulus was presented for 2 seconds, followed by a blank interval lasting 0.5–5 seconds to improve design efficiency. Four characters were added as fillers in the beginning and another four in the end of the study list to eliminate the primacy and recency effects. M: massed learning; S: spaced learning.

Figure 1 illustrates the materials and experimental design. In total, 264 Korean characters were used in this study. Sixty characters were studied under the massed learning condition and another 60 characters were studied under the spaced learning condition, counterbalanced across the participants (i.e., half of the subjects studied set A of the characters in the massed condition and set B in the spaced learning, and the other half did the opposite). Another 120 characters were used as foils in the recognition memory test. To minimize primacy and recency effects, 24 characters (8 for each session) were added in the beginning and the end of the study list. They were excluded from behavioral and fMRI analyses.

### fMRI Task

Subjects lay supine on the scanner bed, and viewed visual stimuli back-projected onto a screen through a mirror attached to the head coil. Foam pads were used to minimize head motion. Stimulus presentation and timing of all stimuli were achieved using E-prime (Psychology Software Tools, Inc. Pittsburgh, PA) on an IBM-compatible PC. During the scan, participants were explicitly instructed to intentionally memorize each character presented on the screen and were also told that a memory test would be conducted after the scanning session. An event-related design was used in this study, with spaced learning and massed learning conditions pseudo-randomly mixed. Each character was repeated four times. In the massed learning condition, the four repetitions of a given character were grouped together with 0 inter-repetition interval. In contrast, in the spaced learning condition, the four repetitions were randomly spaced, with an averaged inter-repetition interval of 12 stimulus presentations, ranging from 6 to 20. For each trial, the stimulus was presented for 2 sec, followed by a random jitter (i.e., fixation) that lasted from 0.5 to 5 sec (mean: 1.5 sec) to improve design efficiency [48]. To avoid primacy and recency effects, four characters were placed in the beginning and another four characters the end of the sequence. They were treated as fillers and encoded as nuisance variables in fMRI data analysis. Participants completed three sessions of the memory task, each lasting 580 sec. In each session, 20 characters were studied under the spaced learning condition and 20 were studied under the massed learning condition.

### Postscan Behavioral Test

A recognition memory test was administered 1 hour after the scan to assess participants' memory. During the recognition memory test, a total of 240 characters (half learned, half new) were randomly mixed together. For each stimulus, the subjects were asked to decide whether it had been learnt on a 6-point confidence scale, with 1 indicating “definitely new” whereas 6 indicating “definitely old”. The stimulus remained on the screen until a response was made. The next item appeared after a 1 sec delay. There was no time pressure for participants to finish the memory test.

### MRI Data Acquisition

Imaging data were acquired on a 3.0 T Siemens MRI scanner in the MRI Center at Beijing Normal University. A single-shot T2*-weighted gradient-echo, EPI sequence was used for functional imaging acquisition with the following parameters: TR/TE/θ = 2000ms/30ms/90°, FOV = 200×200mm, matrix = 64×64, and slice thickness = 4mm. Thirty contiguous axial slices parallel to the AC-PC line were obtained to cover the whole cerebrum and partial cerebellum. Anatomical MRI was acquired using a T1-weighted, three-dimensional, gradient-echo pulse-sequence (MPRAGE). The parameters for this sequence were: TR/TE/θ = 2530ms/3.39ms/7°, FOV = 256×256mm, matrix = 256×256, and slice thickness = 1.33mm. One hundred and twenty-eight sagittal slices were acquired to provide high-resolution structural images of the whole brain.

### Behavioral Data Analysis

Receiver Operating Characteristic (ROC) analysis was conducted on memory performance, separately for spaced and massed learning conditions [49]. In order to correlate behavioral performance with fMRI responses (see below), two behavioral indices were used to describe memory performance. The first index was the number of correct hits with high confidence (scored 5 and 6 on the 6-point scale). Since this result was biased by individuals' response criteria [50], another unbiased discriminability index (d′) was computed using the following formula: d′ = Z_(hit rate)_−Z_(false alarm)_, where hit and false alarm were respectively defined as old and new items that scored 5 and 6. For both indices, paired-sample t-tests were conducted to examine the effect of learning condition (Spaced vs. Massed) on memory performance.

### Image Preprocessing and Statistical Analysis

Image preprocessing and statistical analysis were carried out using FEAT (FMRI Expert Analysis Tool) version 5.98, part of the FSL (FMRIB software library, version 4.1, www.fmrib.ox.ac.uk/fsl). The first three volumes before the task were automatically discarded by the scanner to allow for T1 equilibrium. The remaining images were then realigned to correct for head movements [51]. Translational movement parameters never exceeded 1 voxel in any direction for any subject or session. Data were spatially smoothed using a 5-mm full-width-half-maximum (FWHM) Gaussian kernel. The spatially smoothed data were then filtered temporally using a non-linear highpass filter with a 60-s cut-off. A two-step registration procedure was used whereby EPI images were first registered to the MPRAGE structural image, and then into the standard MNI space, using affine transformations [51]. Registration from structural images to the standard space was further refined using FNIRT nonlinear registration [52], [53]. Statistical analyses were performed in the native image space, with the statistical maps normalized to the standard space prior to higher-level analyses.

Two general linear models within the FILM module of FSL were used to model the data. The first model examined the neural mechanisms of repetition suppression, subsequent memory and the spacing effect. Items were separately modeled according to their repetition condition (Repetition 1 to 4), subsequent memory test outcome (Remembered vs. Forgotten), and learning condition (Massed vs. Spaced). Only characters that were recognized with high confidence scores (5 and 6 on the confidence scale) were considered as Remembered items [32]. Only characters that were judged as new with high confidence scores (1 and 2) were considered as Forgotten items. Items with a score of 3 or 4 (i.e., low confidence) were encoded as a nuisance variable. The first and last four filler characters were also encoded as a nuisance variable. Null events were not explicitly modeled, and therefore constituted an implicit baseline. For each subject, 7 contrast images were computed, including the main effects of repetition suppression (RS: 1^st^ Rep – Rep 2–4), subsequent memory effect (SM: Remembered- Forgotten) and spaced learning (SL: Spaced - Massed), and their 2- and 3-way interactions.

The second model examined individual differences in encoding-related brain activation and their association with subsequent memory performance. All characters, regardless of being remembered or forgotten in the memory test, were encoded as one variable, separately for each repetition and each learning condition. Two contrasts, the overall activation (i.e., [1 1 1 1]) and the amplitude of neural repetition suppression (i.e., [3 −1 −1 −1]), were separately computed for massed learning and spaced learning. These activities were then correlated with individuals' behavioral performance (d′).

Using a fixed-effects model, the higher-level analyses created cross-run contrasts for a set of contrast images for each subject. These contrast images were then input into a random-effects model for group analysis, using ordinary least squares (OLS) estimation. For the second model, individuals' discriminability index (d′) was added as a covariant to examine the relationship between encoding-related brain activities and individuals' subsequent memory performance. Group images were thresholded using cluster detection statistics, with a height threshold of *Z*>2.3 and a cluster probability of *p*<0.05, corrected for whole-brain multiple comparisons using Gaussian Random Field Theory (GRFT).

### Conjunction Analysis

To examine whether spaced learning could reduce repetition suppression in the same regions that were important for memory of novel characters, we then performed a conjunction analysis to examine if there were overlapping neural substrates for spaced learning, repetition suppression, and subsequent memory, using the procedure suggested by Nichols et al. [54]. Group maps for each contrast were thresholded individually at z = 2.3 (corrected for multiple comparisons at the whole-brain level), binarized, and multiplied, which resulted in a map containing brain regions shared by spaced learning, repetition suppression, and subsequent memory.

### Regions of Interest Analysis

Group analyses revealed a significant subsequent memory effect but no significant interactions between learning condition (spaced and massed) and subsequent memory effect (see Results for details) in the left midfusiform, left inferior frontal lobe, and bilateral superior parietal lobule. These regions thus represented common and unbiased regions of interest (ROI) that are responsible for successful memory encoding under both massed and spaced learning conditions. Subsequent ROI analyses were done to examine the effect of spacing on repetition suppression and overall activity in these regions. ROI analyses were performed by extracting parameter estimates (betas) of each event type from the fitted model and averaging across all voxels in each cluster for each subject. Percent signal changes were calculated using the following formula: [contrast image/(mean of run)]×ppheight×100%, where ppheight is the peak height of the hemodynamic response versus the baseline level of activity [55].

To evaluate the correlation between individuals' overall encoding-related brain activation and memory performance, the results were also thresholded using cluster detection statistics, with a height threshold of *Z*>2.3 and a cluster probability of *p*<0.05, corrected for whole-brain multiple comparisons using Gaussian Random Field Theory (GRFT). To further explore the correlational results and to confirm that the correlation was not driven by outliers, a non-independent ROI of the left midfusiform region showing the most significant correlation with memory performance was defined by growing a 6 mm diameter sphere (117 voxels) around the local maxima. The average activation within this sphere was then extracted and plotted against memory performance.

## Results

### Behavioral Data: Spaced Learning Enhanced Subsequent Memory

**Figure 2:**
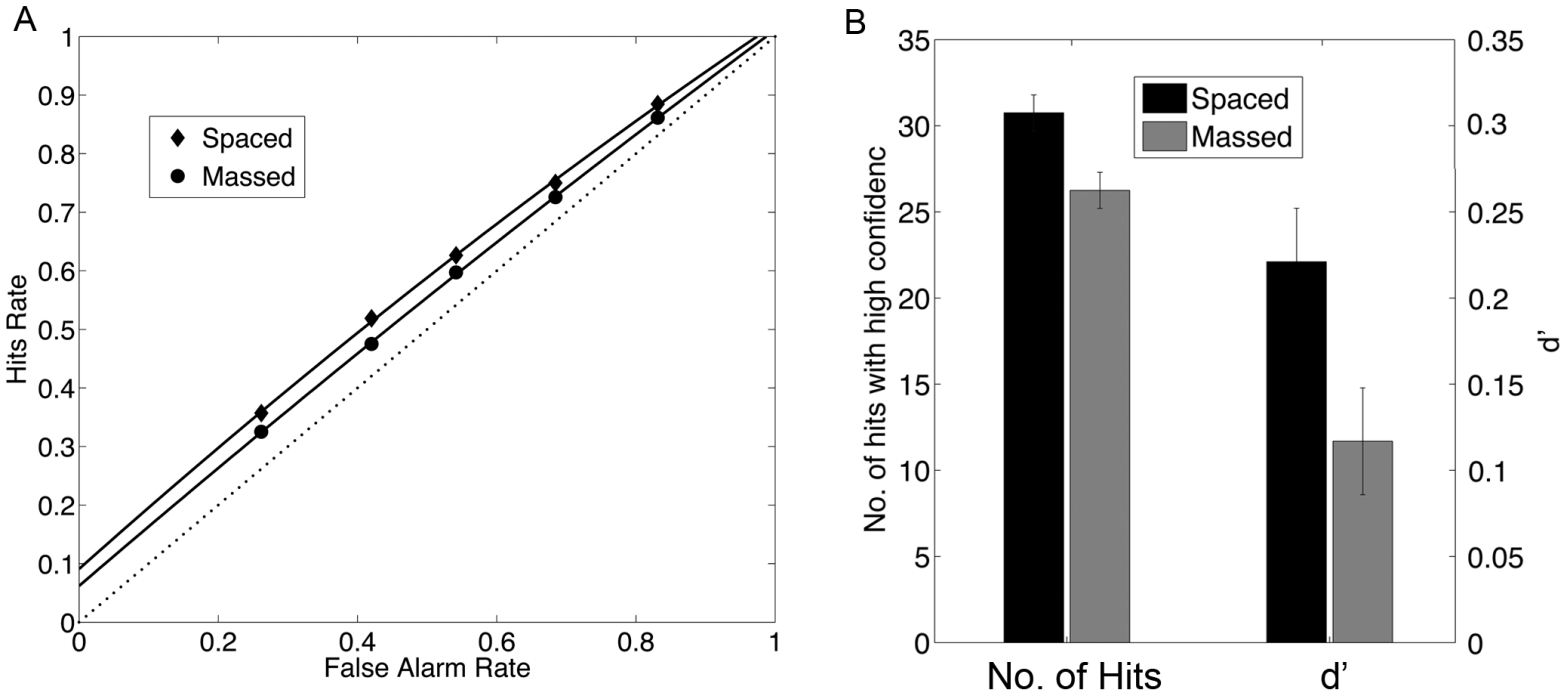
Behavioral effects of spaced learning. A. Receiver operator characteristic (ROC) curve plotted as hits rate against false alarm rate, separately for spaced and massed learning. B. Bars represent the mean number of hits with high confidence (rated as > = 5 in a 6-point scale with 1 indicating definitely new and 6 indicating definitely old), and the mean discriminablity index (d′) for spaced and massed learning in a recognition memory test administered 1 hour after the scan. Error bars represent within-subject standard error.

First, we examined whether spaced learning resulted in any behavioral advantages in recognition memory. Because of the use of novel, nonverbal material as well as the use of highly similar stimuli as fillers in the test, the overall subsequent memory performance was low (Figure 2A). The comparison between the spaced and massed conditions was nevertheless consistent with existing studies: Spaced learning was associated with more overall hits (scored 4 and above, 62% vs. 58%, t(18) = 2.80, p = 0.01) and hits with high confidence (scored 5 and above, 51% vs. 46%; t(18) = 2.70, p = .014). The discriminability index (d′) was also significantly greater under the spaced learning condition than under the massed learning condition (t(18) = 2.75, p = .013) (Figure 2B). This is true even after removing 4 subjects whose overall performance was near chance (i.e., d′< = .05, t(13) = 2.81, p = .014 ). Because d′ was unaffected by individuals' decision criteria, it was then used to correlate with individuals' BOLD activations during learning.

### Repeated Presentation Was Associated with Reduced Neural Activity in the Left Midfusiform

**Figure 3:**
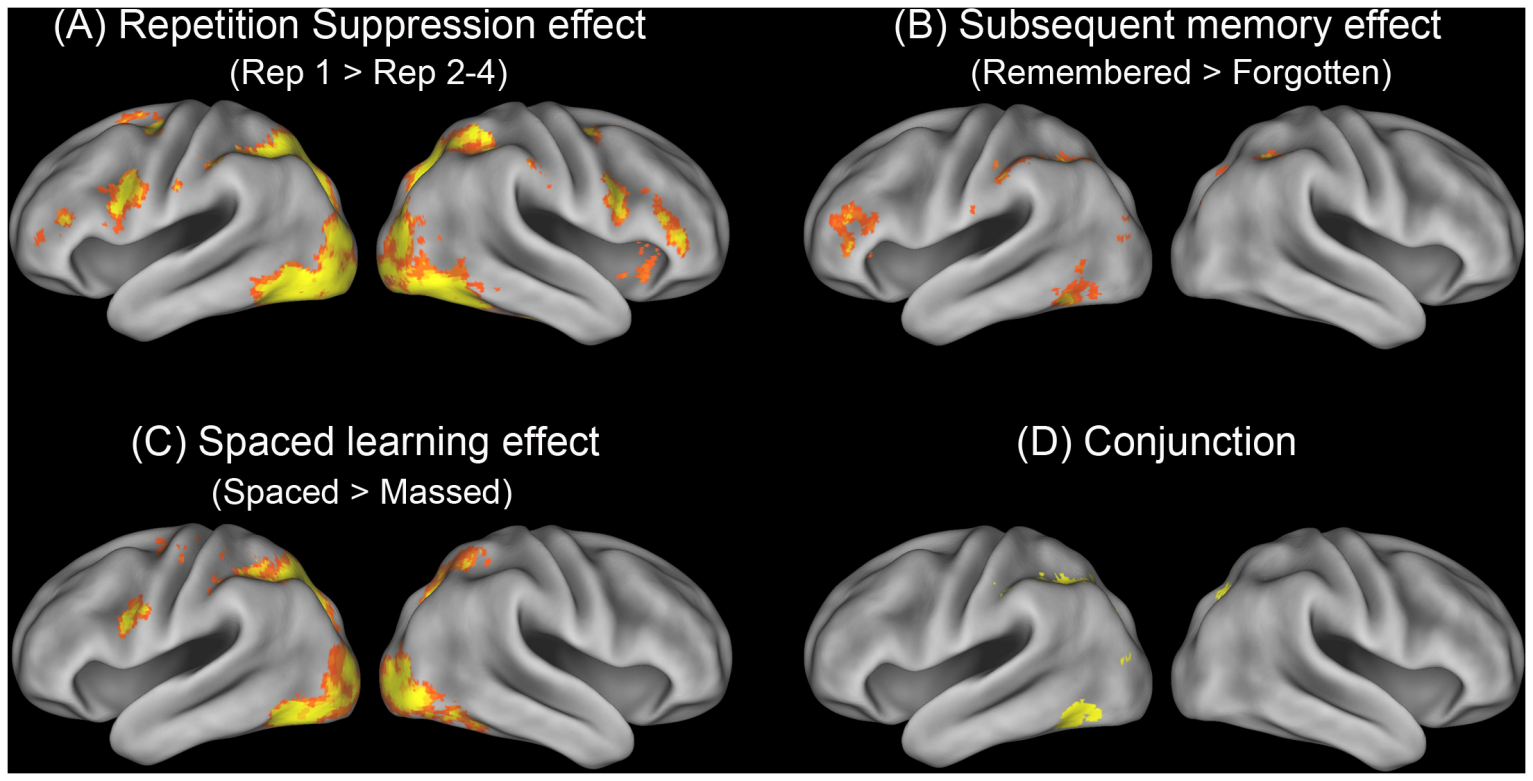
Neural effects of (A) repetition suppression, (B) subsequent memory, (C) spaced learning, and (D) their conjunction. Repetition suppression was assessed by comparing the BOLD responses to the first and subsequent presentations (1^st^ Rep>Rep 2–4). Subsequent memory effect was examined by comparing the neural activity associated with subsequently remembered items with subsequently forgotten items (Remembered>Fogotten). Spacing effect was examined by comparing the BOLD responses to items in the spaced learning condition and those in the massed learning condition, across 4 repetitions (Spaced>Massed). Similar results were obtained by only comparing repetitions 2–4 between the two conditions. All activations were thresholded at z>2.3 (whole-brain corrected) and rendered onto a population-averaged surface atlas using multifiducial mapping [65].

To test our first hypothesis, we compared the BOLD responses to the first and subsequent repetitions. The comparison revealed a strong repetition suppression effect in the bilateral ventral and dorsal ventral stream, including the left (MNI: −44,−66,−8, Z = 4.19) and the right (MNI: 42,−58,−20, Z = 3.70) midfusiform cortices, the bilateral inferior and superior occipital gyri, and the superior parietal lobule. In addition, the bilateral inferior frontal gyrus/precentral gyri, the bilateral frontal pole, the paracingulate cortex, the right putamen, and the bilateral thalamus also showed a significant repetition suppression effect. (Figure 3A, Table 1). These results support our first hypothesis.

**Table 1:** Brain regions showing significant repetition suppression effect (first rep>rep 2–4).

Regions	Volume	x	y	z	Z
Right midfusiform gyrus/inferior occipital gyrus	11201	−26	−84	−12	5.86
		46	−68	−12	5.74
Right superior occipital gyrus/superior parietal lobule		32	−70	24	5.47
		32	−52	40	4.61
Left midfusiform gyrus/inferior occipital gyrus	10018	−46	−62	−10	6.01
		−48	−70	−6	5.43
Left superior occipital gyrus/superior parietal lobule		−14	−66	−46	5.41
		−32	−58	54	4.82
Right precentral gyrus/inferior frontal gyrus	1283	50	10	26	4.46
Left precental gyrus/inferior frontal gyrus	990	−46	4	28	4.71
Left inferior frontal gyrus	311	−40	26	12	3.74
Right precental gyrus/middle frontal gyrus	376	30	2	50	4.00
Left precental gyrus/middle frontal gyrus	1978	−28	−6	44	5.89
Paracingulate gyrus/SMA		−4	12	48	5.74
Right putamen	374	22	18	−4	3.98
Left thalamus		−6	18	4	3.74
Right thalamus		4	−18	4	3.70

### The Left Midfusiform Supported Memory of Novel Characters

To test our second hypothesis regarding the association between left midfusiform activity and episodic memory for novel characters, we first examined whether the midfusiform activity could predict remembered vs. forgotten characters within subjects, using the subsequent memory approach [30], [31]. Comparing the subsequently remembered items (high-confidence hits) with the subsequently forgotten items (high-confidence rejects) revealed significant activations in the left midfusiform gyrus (MNI: −48,−58,−16, Z = 3.79). Strong activations were also found in the left (MNI: −34,48,32, Z = 4.13) and the right (MNI: 36, −48, 44, Z = 3.61) superior parietal lobules that extended to neighboring angular and supramarginal gyri, as well as in the left inferior frontal gyrus (MNI: −34, 30, 6, Z = 3.92) that extended to the frontal pole (Figure 3B, Table 2). No significant interaction between learning condition and subsequent memory effect was found in these regions, suggesting that these regions were important for successful memory encoding in both the spaced and massed learning conditions.

**Table 2:** Brain regions showing significant subsequent memory effect (remembered>forgotten characters).

Regions	Volume	x	y	z	Z
Left superior parietal lobule/angular gyrus/supramarginal gyrus	1502	−34	−48	32	4.13
Right superior parietal lobule/angular gyrus/supramarginal gyrus	1049	36	−48	44	3.61
Left inferior frontal gyrus/frontal pole	625	−34	30	6	3.92
Left inferior temporal gyrus/midfusiform	469	−48	−58	−16	3.79

**Figure 4:**
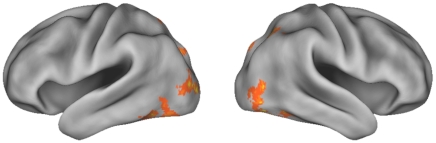
Summed activity predicted individuals' memory performance. Brain regions showing significant correlations (Z>2.3, whole-brain corrected) between summed activity and subsequent memory (d′), are rendered onto a population-averaged surface atlas using multifiducial mapping [65].

We then examined whether individual variations of brain activity in these regions could predict subsequent memory performance. By correlating individuals' memory performance (d′) with brain activities during learning, we found significant positive correlations in the left midfusiform cortex (xyz in MNI: −44,−56,−12, Z = 4.61), which extended to the adjacent left inferior temporal gyrus (Figure 4; Figure S1). It should be emphasized that the local maxima of the left midfusiform cortex revealed in the cross-subject correlation analysis overlapped with that showing within-subject subsequent memory effect (i.e., −48 −58, −16). Other regions showing positive correlations included the bilateral dorsal and ventral lateral occipital cortices (Table 3).

**Table 3:** Brain regions showing significantly positive cross-subject correlation between summed activation and subsequent memory performance (d′).

Regions	Volume	x	y	z	Z
Left midfusiform gyrus/inferior temporal gyrus	1233	−44	−56	−12	4.61
Left dorsal lateral occipital cortex	694	−14	−86	36	4.05
Left ventral lateral occipital cortex	612	−26	−90	4	4.71
Right inferior occipital cortex	1314	42	−70	−16	4.63
Right dorsal lateral occipital cortex	726	34	−66	26	4.15

### Spaced Learning Reduced Repetition Suppression in Regions Associated with Memory of Novel Characters

Having identified the regions important for memorizing novel characters, we then examined our third hypothesis, that is, whether spaced learning could reduce neural repetition suppression in these regions. A comparison of the spaced learning condition with the massed learning condition revealed significant activation in the left fusiform cortex and the inferior occipital cortex (IOC, MNI: −38,−58,−12, Z = 5.3), the right fusiform/IOC (MNI: −46,−84,−8, Z = 5.05), the bilateral superior occipital gyrus that extended to superior parietal lobule (Left: −24,64,42, Z = 5.29; Right: 30,−66,36, Z = 4.73), as well as the precentral gyrus/IFG (MNI: −48,4,26, Z = 4.3) (Figure 3C, Table 4). Essentially, conjunction analysis revealed that spaced learning significantly enhanced activity in the same regions important for subsequent memory, including the left midfusiform cortex and the bilateral superior occipital gyrus/superior parietal lobule (Figure 3D, Table 5). In addition, these exact regions also showed significant repetition suppression effect.

**Table 4:** Brain regions showing significant spacing effect (Spaced learning>massed learning).

Regions	Volume	x	y	z	Z
Left midfusiform/inferior occipital gyrus	7008	−38	−58	−12	5.3
		−38	−74	−12	4.89
Left superior occipital gyrus/superior parietal lobule		−24	64	42	5.29
		−38	−54	50	4.70
Right midfusiform gyrus/inferior occipital gyrus	4649	−46	−84	−8	5.05
		42	62	14	3.99
Right superior occipital gyrus/superior parietal lobule		30	−66	36	4.73
		40	−48	56	3.73
Left precentral gyrus/inferior frontal gyrus	533	−48	4	26	4.3
	333	−24	−12	50	3.82

**Table 5:** Brain regions showing conjunctive effect of spaced learning, repetition suppression and subsequent memory.

Regions	Volume	x (COG)	y (COG)	z (COG)
Left superior occiptal gyrus/superior parietal lobule	719	−34	−56	40
Right superior occiptal gyrus/superior parietal lobule	412	30	−64	36
Left midfusiform gyrus/inferior temporal gyrus	299	−44	−58	−16

COG: Center of gravity.

**Figure 5:**
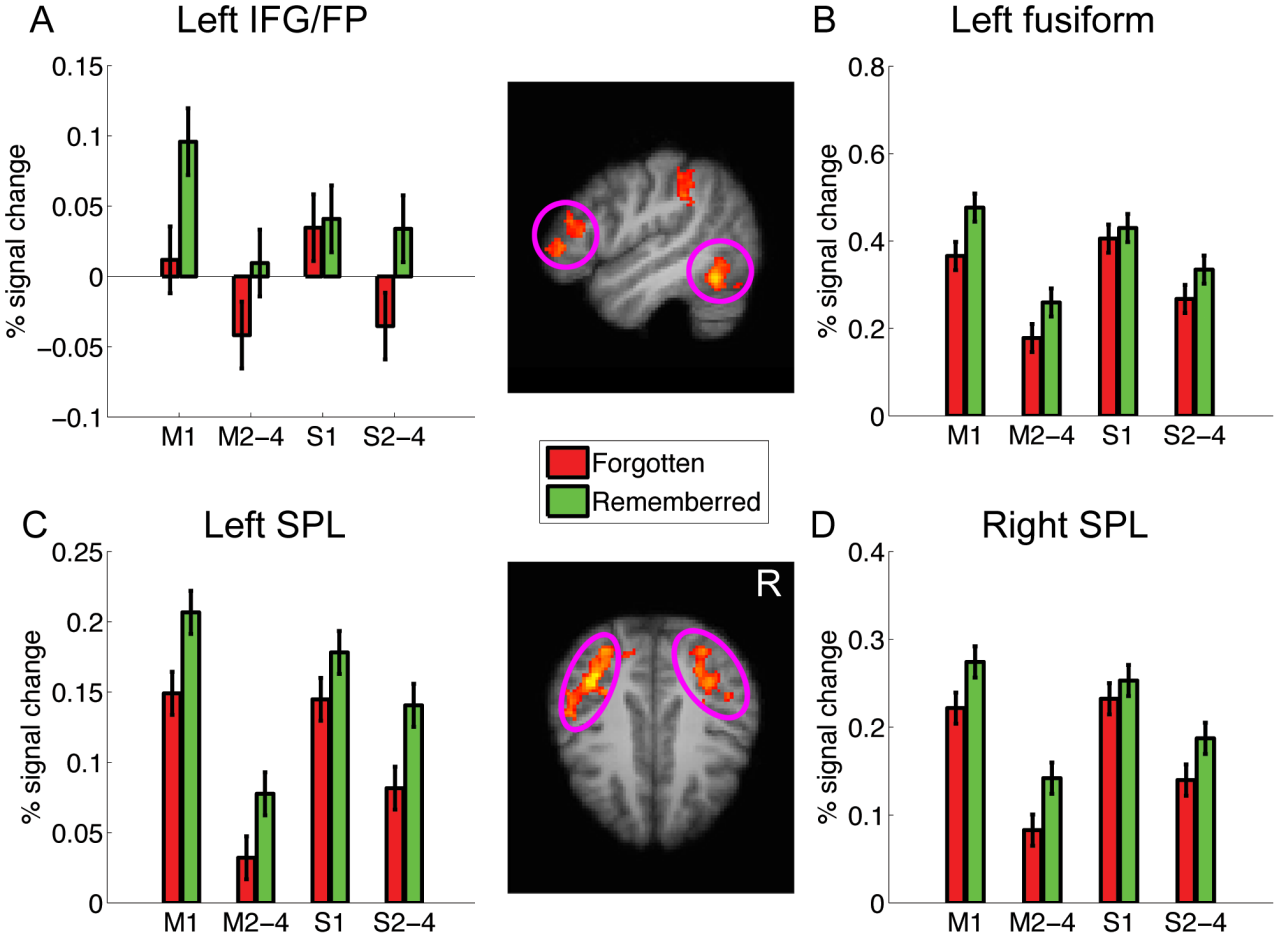
Spaced learning enhanced neural activity associated with memory encoding. Activation map represents brain regions showing a significant subsequent memory effect, thresholded at z>2.3 (whole-brain corrected), which are overlain on the sagittal (top) and axial (bottom) slices of the group mean structural image. (A–D) Plots of percentage signal change in each ROI, separately for the first presentation and the following repetitions. Error bars denote within-subject standard error.

We then examined the hypothesis that spaced learning enhanced neural activation by reducing repetition suppression. We extracted the BOLD signal changes for each condition from regions showing the subsequent memory effect, including the left middle frontal gyrus (LMFG), the left midfusiform (Lfus), and the bilateral superior parietal lobule (SPL) (Figure 5). First, we found that neural activity during the first presentation did not differ significantly between the spaced and massed conditions (Fs<1). Second, three-way repeated measures ANOVA analysis revealed significant interactions between repetition priming and spaced learning in the left midfusiform gyrus (F(1,18) = 5.21, p = .035), and the left (F(1,18) = 7.80, p = .012) and the right SPL (F(1,18) = 6.841, p = .0175), but not the LMFG ( F(1,18) = 1.19, p = .29), suggesting that spaced learning significantly reduced repetition suppression in the former three regions. These results confirmed the hypothesis that spaced learning could enhance memory for novel characters and reduce neural repetition suppression in the brain regions that supported subsequent memory.

## Discussion

The present study examined and confirmed three hypotheses regarding the role of the left midfusiform in processing and memorizing novel characters. First, we found that repeated visual exposure to novel characters was associated with decreased neural activation in the left midfusiform cortex, along with decreases of neural activities in several other regions in the dorsal and ventral visual stream and the inferior frontal gyrus. Second, activation in the left midfusiform cortex predicted memory for novel characters both across and within subjects (i.e., across items), with stronger midfusiform activation associated with better recognition. More importantly, by manipulating repetition lag, we showed that spaced learning increased learning-related neural activity in the left midfusiform cortex and also enhanced memory for novel characters. These results demonstrated a strong link between activity of the left midfusiform cortex and memory for novel characters, which has important implications for the visual word form area (VWFA) hypothesis as well as the neural mechanisms of the spacing effect.

Although researchers agree on the important role of the left midfusiform in reading, the specific role it plays and its developmental mechanisms still remain controversial. According to one theoretical account, the left midfusiform cortex is the visual word form area (VWFA) [1], [2], specialized for the processing of the visual form of familiar words [14]. Because written language is a relatively recent cultural invention and the human brain is not born with the capacity to read, the VWFA is developed by “invading” the evolutionarily older brain circuits that support general object recognition [56], and with enhanced perceptual mechanisms acquired via extensive visual experience with specific sets of written words [28]. Evidence from other research suggests a different hypothesis, that is, the midfusiform is not specialized for visual word forms [15], [16], [18], rather, it is developed through the learning of multiple, interactive visual and linguistic components [16], [17]. In particular, orthographic learning leads to decreased rather than increased midfusiform activation [16], [17], [18].

Our data are consistent with the latter hypothesis. First, in line with several previous observations [16], [17], [18], [20], [57], we found a strong midfusiform response to foreign characters with which participants were not familiar. This result is also consistent with several other studies that found significant differences between native and foreign writing in the more anterior fusiform region but not in the midfusiform cortex [19], [21]. This suggests that the functional localizer paradigm used in these studies, although useful in identifying word-sensitive regions outside the midfusiform, might have missed the regions within the fusiform territory that are important for the processing and memorization of foreign characters (See below).

Second, we found that strong activations in this region supported recognition memory for novel characters, in a way similar to memory for familiar words [31]. From both within- and cross-subject analyses, we found that weaker midfusiform activation during learning was associated with worse recognition memory. Spacing the repetitions of study materials reduced neural repetition suppression in the left midfusiform cortex and therefore increased the overall learning-related activity, and also enhanced the long-term memory of the novel characters. This corroborates our existing results [24], [25], [26] and further supports the important role of the midfusiform in learning and memorizing new characters. Interestingly, the midfusiform is found to be important for face memory [29], [58], which further challenges the VWFA hypothesis.

Third, we found significant reduction of neural activation in the left midfusiform as a result of repeated exposure to novel characters, under both the massed and spaced learning conditions. These results and those found in another study of learning novel faces [29] did not replicate the previous results showing increased neural activity associated with repetition of novel stimuli [27]. Previous studies on long-term orthographic training [16], [18] have also found neural activity reduction in the left midfusiform gyrus. Similar results have been found in other types of visual perceptual training, including musical notation [59]. To explain the observed increase of midfusiform activation for familiar words in comparison to nonwords, we have proposed that such increased activation might have resulted from the associations between visual form and other linguistic factors, such as phonology and semantics [16], [17], [18].

Our results also shed new light on the neural mechanisms underlying the spacing effect in long-term memory. This effect has been revealed using various learning tasks and materials [60], [61], [62]. Specifically, studies using novel nonverbal materials, such as nonsense shapes [63], unfamiliar faces [34], [40] and nonwords [34], [35], [36], have found that spaced learning can enhance memory by reducing short-term perceptual priming. That is, stronger perceptual priming under the massed presentation condition leads to reduced perceptual processing of targets in their second and later presentations, and hence worse performance in the cued-recognition test that relies on the retrieval of the structural-perceptual information about the targets.

We found that spaced learning reduced repetition suppression and increased the overall processing strength in the left midfusiform cortex, which in turn were associated with better recognition memory. These results are thus consistent with the idea that repetition suppression hinders episodic memory [29], [33]. The neural evidence from the present study is also consistent with the deficient processing hypothesis in general [37], [44] and the short-term perceptual priming hypothesis in particular [40]. One major difference between the present study and those using familiar words as study material lies in the locus of the spacing effect. The latter studies found that the spacing effect was primarily mediated by activity in the inferior frontal gyrus [33], [44]. The absence of the frontal effect in our study suggests that enhanced subsequent memory as a result of spacing in our study may not be a result of top-down modulation from prefrontal cortex [64], but may indeed reflect greater perceptual encoding (i.e., bottom-up processing). The current study also controlled the voluntary attention effect by using an intentional memory encoding task, in which subjects were asked to memorize each item and were informed in advance about the memory test. One limitation of this paradigm was a lack of behavioral indices during learning, which prevented us from examining the behavioral repetition priming effect and monitoring the attention state of the subjects during learning.

Although behavioral and fMRI results from the spacing effect support the idea that repetition priming could hinder recognition memory by reducing encoding-related processing and brain activity, quantitative examinations of the relationship between repetition priming and subsequent memory have failed to reveal any strong negative correlation either within or across subjects [29], [45]. This is also the case in the present study. Still other studies found that stronger repetition priming was associated with better subsequent memory [46]. We have proposed that this discrepancy could be resolved by considering factors such as the variance in stimuli that could affect repetition priming [29]. Consistent with this view, when there was no difference in brain activity between remembered and forgotten items in the first presentation (suggesting a good control of variance in stimuli), there was a significant relation between repetition priming and subsequent memory [29]; when such difference in stimuli was present, which was the case in the present study, the relation between repetition priming and subsequent memory was not significant (although in the expected direction). The results from these two studies based on similar paradigms thus add new evidence to the above view and call for future studies to examine this issue. Moreover, future studies need to establish causal relations between neural repetition suppression and the spacing effect, perhaps by examining whether controlling neural repetition suppression could eliminate the behavioral spacing effect [34], [35].

In summary, our study shows that better memory for novel characters can be achieved by increasing neural activation of the left midfusiform using a spaced learning paradigm. In addition to further examining the neural mechanisms underlying the spacing effect, future studies need to examine whether other strategies that reduce neural repetition priming could also increase memory performance. Moreover, future studies need to examine whether these mechanisms can be applied to daily-life and classroom learning situations and to other aspects of learning to read, such as form-sound association, and form-meaning association. Results from such studies will have potential educational implications.

## Supporting Information

Figure S1 Summed activity predicted individuals' memory performance. (A) Brain regions showing significant correlations (Z>2.3, whole-brain corrected) between summed activity and subsequent memory (d′), are overlain on the axial slice of the group mean structural images. (B) Scatterplot activation in the left fusiform cortex versus memory performance. Please note that the ROI is not defined independently, and the scattorplot is only to show that the correlation is not driven by outlier (s).
(0.25 MB TIF)
