# Supplementary figures and images for: Facilitating Memory for Novel Characters by Reducing Neural Repetition Suppression in the Left Fusiform Cortex
Source: PLoS One. 2010 Oct 6;5(10):e13204. doi: 10.1371/journal.pone.0013204 (PMC2950859; doi:10.1371/journal.pone.0013204)

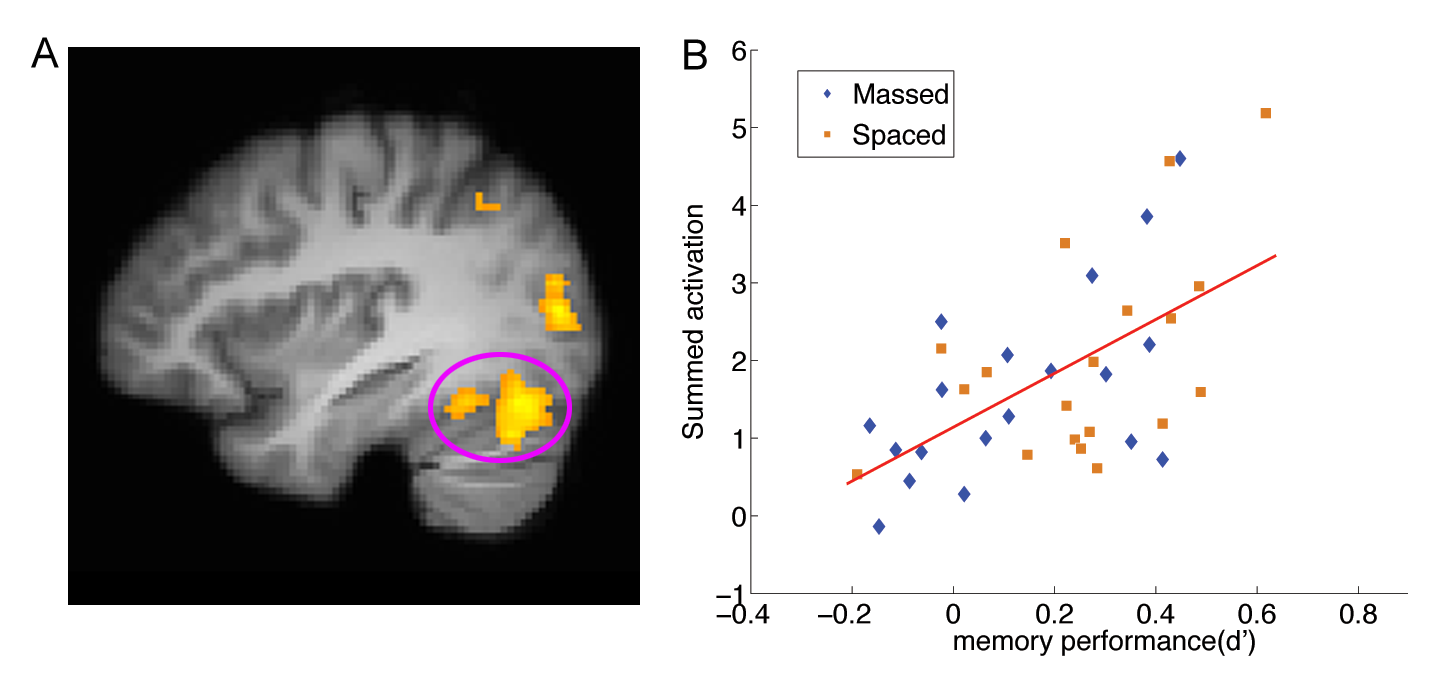

Supplement: Figure S1 — Summed activity predicted individuals' memory performance. (A) Brain regions showing significant correlations (Z>2.3, whole-brain corrected) between summed activity and subsequent memory (d′), are overlain on the axial slice of the group mean structural images. (B) Scatterplot activation in the left fusiform cortex versus memory performance. Please note that the ROI is not defined independently, and the scattorplot is only to show that the correlation is not driven by outlier (s). (0.25 MB TIF) [file pone.0013204.s001.tif]
